# BK Virus and Nephropathy in Kidney Transplant Recipients

**Published:** 2016-11-01

**Authors:** B. Einollahi, I. Lotfian

**Affiliations:** 1 *Nephrology and Urology research Center, Baqyiatallah University of Medical Science, Tehran, Iran*; 2 *Health Research Center, Baqiyatallah University of Medical Sciences, Tehran, Iran*

**DEAR EDITOR**

Polyomavirus-associated nephropathy (PVAN) remains an important cause of allograft dysfunction and graft loss after kidney transplantation [1, 2]. Furthermore, up to 10% of renal transplant recipients show a viral reactivation that can lead to PVAN and delayed treatment results in high risk of graft loss (up to 80%) [3]. We read with interest the article published by Pakfetrat, *et al* [2], and would like to comment on that.

BKV infection can be detected by viral serology, urine cytology, or nucleic acid testing methods such as quantitative PCR (Q-PCR) analysis of urine, plasma or blood samples [1, 2]. Viral load monitoring in urine and plasma by real-time PCR after transplantation is the most common diagnostic tool to detect viral reactivation [3]. Based on the results of large cross-sectional and prospective longitudinal studies using nucleic acid testing for the detection of BKV reactivation after renal transplantation, it seems that, with time, approximately 5%–15% of patients become BKV-positive in their plasma and 20%–40% of patients become BKV-positive in their urine [1]. Progression of BK viruria to PVAN without concomitant viremia is exceptionally rare [1]. There are some protocols for screening BKV. Several investigators suggest to first check the viremia, while others recommend checking viruria [1, 4]; however, all experts agree on the need to combine both plasma and urine determinations of the BKV DNA load in order to identify renal transplant patients at high risk of BKV-associated nephropathy [5]. In addition, the screening of kidney transplant recipients with nucleic acid testing of plasma or urine, has been controversial still [4].

In the lack of standardized Q-PCR assays for BKV, clinical and research laboratories have developed their own in-house Q-PCR methods using various primers, probes and standards. As a result, making comparisons of BKV loads among laboratories is difficult and guidelines for quantitative cut-off values for viral load in urine and plasma are only of limited use in screening strategies or monitoring protocols [1].

In 2005, guidelines for BKV screening using nucleic acid testing were formulated and included quantitative cut-off values for viral loads as a threshold for performing additional testing. The guidelines stated that if urine viral loads exceeded 10^7^ copies/mL or if plasma viral loads were higher than 10^4^ copies/mL for more than three consecutive weeks, a renal biopsy should be considered [1].

Diagnostic cut-off values for PVAN in urine samples varying between 7.9×10^6^ and 2.5×10^7^ copies/mL, and plasma cut-off values between 3.0×10^3^ and 3.2×10^4^ copies/mL, clearly illustrate the inter-assay variability [1]. The observed substantial inter-assay variability occurs for a number of reasons—differences in sample type (urine, serum, plasma, and blood), differences in DNA extraction methods, different features of primer and probe design (including effects of BKV subtype-associated polymorphisms), different amplification conditions, and different choices of reference material (*e.g.*, mixed-patient standard *vs* Dunlop strain genome plasmid) [1].

Finally, we completely agree that screening methods and detection of BK virus and BKVN are different and further studies to standardize and uniform the protocols are needed.
