# Supplementary material for: BK Virus and Nephropathy in Kidney Transplant Recipients
Source: Int J Organ Transplant Med. 2016 Nov 1;7(4):238–40. (PMC5219586)
Supplement: Related texts

# Authors’ Reply

**Published:** 2016-11-01

**Authors:** R. Yaghobi

**Affiliations:** 1 *Shiraz Transplant Research Center, Namazi Hospital, Shiraz University of Medical Sciences,Shiraz, Iran*

**DEAR EDITOR,**

Polyomavirus BK and its related clinical complications, especially BKVAN, are among the important viral complications with roles in the pathogenesis of nephropathy in kidney transplant recipients. Our center has focused on this subject to evaluate and study different basic and clinical aspects of BK virus pathogenesis. Our article is the first report on this issue. In our report, we only evaluated a small group of transplant recipients who suspected with BKVAN by evaluating BK viral load in plasma and FFPE tissue samples taken from the patients. In our report, the presented cut-off value for plasma and tissue load of BK viral genome were not used to confirm direct correlation between BK viral active infection and BKVAN. The cut-off value was used as a predictive value to screen the suspected patients from others for future follow-up. However, the results need to be confirmed and standardized in further studies.

Before introduction of noninvasive diagnostic tests for polyomavirus BK replication, BKVAN was mostly diagnosed in advanced stages based on histological examination of the kidney biopsy specimens [1]. In these years, molecular methods including PCR and real-time PCR have significantly contributed to rapid accurate diagnosis of polyomavirus BK [2], and targeted reduction of immunosuppression to resolve infection and improve renal function [3, 4]. Screening for BK virus infection in urine or plasma samples taken from patients, is a matter of debate; controversy exists on importance of each of them in evaluation of viral pathogenesis. The importance of molecular analysis of BK viruria and urine cytology for the diagnosis of BKVAN is clearly mentioned in earlier reports. A negative test result in BK viruria and cytology has a very high negative predictive value (almost 100%). However, a positive test in urine is not associated with an increased risk of BKVAN and requires evaluation of BK viremia in those recipients whose BK viruria exceeds the established thresholds. In addition, Molecular analysis of BK viruria is expensive and time-consuming and needs more follow-up as BK viremia [5]. There is also a direct correlation between BK viremia load, degree of graft dysfunction, severity of histological BKVAN tissue patterns, and number of infected renal cells in histological slides [6]. Such correlation suggests that viremia occurs mainly, if not entirely, from the viral replication started in the kidney. This is the reason why the amount of viremia is more predictive for BKVAN than presence of viruria [7-10]. Therefore, use of real-time PCR protocols to detect BK virus in plasma is now recommended by Kidney Disease: Improving Global Outcomes (KDIGO) [11]. 

On the hand, researchers recently reported marked variability between commonly used polyomavirus BK load assays related to rearrangements occurred in different loci of BK virus genome sequences, especially in NCCR [12, 13]. Therefore, neither polyomavirus BK real-time PCR protocol nor the cut-off value for significant viral replication has so far been completely standardized [1, 14-16]. Few data have been published on the specificity and sensitivity of blood BK virus load measurement for BKVAN screening. In 2005, preliminary recommendations were published proposing that a threshold value of 1.0×104 copies/mL for presumptive BKVAN should be followed by an allograft biopsy [17, 18]. However, Hoffman, et al, and others recently reported marked variability between commonly used BK viral load assays and diagnostic cut-off values [8, 19, 20]. Based on these results, we believe that in the absence of standardization for measurement of blood BK virus load, a threshold value for BKVAN screening should be established for each different molecular assay protocols. 

## References

[1] Chung BH, Hong YA, Kim HG (2012). Clinical usefulness of BK virus plasma quantitative PCR to prevent BK virus associated nephropathy. Transpl Int.

[2] Echavarría M, Basilotta N, Aguiar A (2007). BK virus nephropathy after renal transplantation [Diagnosis and prognosis by real time PCR]. Medicina (B Aires).

[3] De Moura Montagner J, Michelon TF, Schroeder RB (2007). Polyomavirus–an emergent pathogen in transplant recipients. Einstein.

[4] Boudreault AA, Courtemanche C, Latulippe E (2009). Screening for polyomavirus associated nephropathy in renal transplantation with blood viral load measurement. J Clin Virol.

[5] Alméras C, Vetromile F, Garrigue V (2011). Monthly screening for BK viremia is an effective strategy to prevent BK virus nephropathy in renal transplant recipients. Transpl Infect Dis.

[6] Drachenberg CB, Hirsch HH, Papadimitriou JC (2007). Polyomavirus BK versus JC replication and nephropathy in renal transplant recipients: a prospective evaluation. Transplantation.

[7] Si-Mohamed A, Goff JL, Désiré N (2006). Detection and quantitation of BK virus DNA by real-time polymerase chain reaction in the LT-ag gene in adult renal transplant recipients. J Virol Methods.

[8] Hirsch HH, Knowles W, Dickenmann M (2002). Prospective study of polyomavirus type BK replication and nephropathy in renal-transplant recipients. N Engl J Med.

[9] Funk GA, Steiger J, Hirsch HH (2006). Rapid dynamics of polyomavirus type BK in renal transplant recipients. J Infect Dis.

[10] Hymes LC, Warshaw BL (2006). Polyomavirus (BK) in pediatric renal transplants: evaluation of viremic patients with and without BK associated nephritis. Pediatr Transplant.

[11] Kidney Disease: Improving Global Outcomes (KDIGO) Transplant Work Group (2009). KDIGO clinical practice guideline for the care of kidney transplant recipients. Am J Transplant.

[12] Miller S, Liverman CS, Post L (2012). Analytical and clinical performance characteristics of the Simplexa BK virus quantitative PCR assay for the diagnosis of polyomavirus-associated nephropathy in renal transplant recipients using plasma and urine specimens. J Clin Virol.

[13] Al-Jedai AH, Honaker MR, Trofe J (2003). Renal allograft loss as the result of polyomavirus interstitial nephritis after simultaneous kidney-pancreas transplantation: results with kidney retransplantation. Transplantation.

[14] Girmanova E, Brabcova I, Bandur S (2011). Aprospective longitudinal study of BK virus infection in 120 Czech renal transplant recipients. J Med Virol.

[15] Chung BH, Hong YA, Kim HG (2012). Clinical usefulness of BK virus plasma quantitative PCR to prevent BK virus associated nephropathy. Transpl Int.

[16] Boudreault AA, Courtemanche C, Latulippe E (2009). Screening for polyomavirus associated nephropathy in renal transplantation withblood viral load measurement. J Clin Virol.

[17] Hirsch HH, Brennan DC, Drachenberg CB (2005). Polyomavirus-associated nephropathy in renal transplantation: interdisciplinary analyses and recommendations. Transplantation.

[18] Ramos E, Drachenberg CB, Portocarrero M BK virus nephropathy diagnosis and treatment: experience at the University of Maryland Renal Transplant Program. Clin Transpl.

[19] Hoffman NG, Cook L, Atienza EE (2008). Marked variability of BKvirus load measurement using quantitative real-time PCR among commonly usedassays. J Clin Microbiol.

[20] Randhawa P, Ho A, Shapiro R (2004). Correlates of quantitative measurement of BK polyomavirus (BKV) DNA with clinical course of BKV infection in renal transplant patients. J Clin Microbiol.
